# Protective effects of a traditional herbal extract from *Stellaria dichotoma* var. *lanceolata* against *Mycobacterium abscessus* infections

**DOI:** 10.1371/journal.pone.0207696

**Published:** 2018-11-19

**Authors:** Su-Jin Bae, Jae-Won Choi, Byung-Joon Park, Jina Lee, Eun-Kyeong Jo, Young-Ha Lee, Sung-Bae Kim, Jae-Min Yuk

**Affiliations:** 1 Department of Infection Biology, College of Medicine, Chungnam National University, Daejeon, Korea; 2 Department of Medical Science, College of Medicine, Chungnam National University, Daejeon, Korea; 3 Department of Microbiology, College of Medicine, Chungnam National University, Daejeon, Korea; 4 Herbal Medicine Research Division, Korea Institute of Oriental Medicine, Daejeon, South Korea; Universitat Hohenheim, GERMANY

## Abstract

*Stellaria dichotoma* var. *lanceolata* (*SdLv*), a member of the Caryophyllaceae, is a traditional herbal medicine that has been used to treat fever, night sweats, and malaria in East Asia. Inflammation plays an essential role in both host defense and pathogenesis during infection by diverse intracellular pathogens. Herein, we showed that an herbal extract from *SdLv* effectively attenuated inflammatory responses from infection of *Mycobacterium abscessus* (Mab), but not *Toxoplasma gondii* (*T*. *gondii*). In primary murine macrophages, *Mab* infection resulted in the rapid activation of nuclear factor (NF)-κB and mitogen-activated protein kinase (MAPK), as well as in the generation of proinflammatory cytokines, such as tumor necrosis factor α and interleukin-6, which were all significantly inhibited by pretreatment with *SdLv*. However, herbal extracts from *Bupleurum chinense* DC. (*Buch*) or *Bupleurum falcatum* L. (*Bufa*) did not affect *M*. *abs*-induced activation of proinflammatory responses. Importantly, we demonstrated that generation of intracellular reactive oxygen species, which are important signaling intermediaries in the activation of NF-κB and the MAPK signaling pathway, was rapidly increased in *Mab*-infected macrophages, and this was effectively suppressed by pretreatment with *SdLv*, but not *Buch* and *Bufa*. We further found that the treatment of *Buch* and *Bufa*, but not *SdLv*, led to the activation of NF-κB and the MAPK signaling pathway and the generation of intracellular reactive oxygen species. Moreover, oral administration of *SdLv* significantly reduced lethality in *Mab*-infected mice. Collectively, these results suggest the possible use of *SdLv* as an effective treatment for *Mab* infection.

## Introduction

Natural products from plants have been used both clinically and as folk medicines for the treatment of various diseasesand have also been demonstrated to be an important resource of novel lead compounds [1, 2]. *Stellaria dichotoma* L. var. *lanceolota* Bunge (*SdLv*), generally called Yin Chai Hu, is commonly distributed in East Asia, including China and the Republic of Korea, as a staple herbal medicine used to treat fever and infantile malnutrition [3]. Moreover, recent studies reported that components isolated from *SdLv* contribute to various biological functions, including anti-inflammatory [4], anti-allergic [5], and antioxidant activities [6]. *Bupleuri Radix* includes plants in the genus, *Bupleurum*, which are commonly used to treat colds, fever, influenza, hepatitis, and malaria in East Asia [7, 8]. Among them, *Bupleurum chinense* DC. (*Buch*), also called Bei Chai Hu in Chinese, has been used to treat fever, influenza, and malaria [9, 10]. Numerous studies have also indicated that *Buch*-derived compounds, such as saikosaponins, flavonoids, and fatty acids, exhibit diverse physiological functions, including anti-inflammatory, antibiotic, antiviral, and immunomodulatory effects [11, 12]. Additionally, *Bupleurum falcatum* L. (*Bufa*), also called Sandao chaihu in Chinese, is a traditional Asian herbal medicine that improves stress-induced depression and anxiety-like behaviors in rats [13–15].

Ancient records of traditional Chinese medicine practices indicate that the dried roots of *Buch* and *B*. *scorzonerifolium* Willd. were prescribed as *Radix Bupleuri* [16, 17]. Moreover, *Bufa* and its related species are used in Korea as traditional medicines [18]. According to the Encyclopedia of Chinese Materia Medica, because of its similar morphology and name, *SdLv* was used as a substitute for *Radix Bupleuri* [16, 19]. However, because of noticeable differences with *B*. *chinense* in terms of medicinal effects, the latter was continuously used as a new herb and is currently known as *Stellariae Radix* in China. For these reasons, the species of *Radix Bupleuri* and its clinical applications are confusing [16, 17]. Thus, comparative studies to characterize the different effects of the three different species are needed.

Intracellular pathogens have evolved various strategies to invade, survive, and replicate inside a variety of cell types through the evasion of the host immune system [20, 21]. *Mycobacterium abscessus* (*Mab*) belongs to a group of rapidly-growing mycobacterium that cause a broad range of human infections, especially chronic lung disease in elderly patients with bronchiectasis and young patients with cystic fibrosis [22, 23]. Moreover, numerous cases of community- and hospital-acquired *Mab* infections have been widely reported throughout the world [24]. Importantly, *Mab* is known to be resistant to most antibiotics, including first-line anti-tuberculous drugs, resulting in a particularly high mortality rate because of treatment failures [22, 25]. *Toxoplasma gondii* (*T*. *gondii*) is an intracellular protozoan parasite that infects a broad range of warm-blooded animals, including avian and mammalian species [26]. Although *T*. *gondi*-infected healthy patients remain asymptomatic, *T*. *gondii* exposure in immunocompromised and congenitally infected patients may result in toxoplasmosis that can lead to high morbidity and mortality [27].

Pathogen-associated molecular pattern molecules derived from various microorganisms are recognized by pattern recognition receptor (PRR)-bearing cells, which then activate an inflammatory response that can contribute not only to host protective immunity but also to immunopathology [28, 29]. Mitogen-activating protein kinases (MAPKs) and nuclear factor (NF)-κB play important roles in promoting inflammatory responses following pathogen infection [30, 31]. Recent studies have reported that intracellular pathogens, including *Mab* and *T*. *gondii*, strongly induced various proinflammatory cytokines through MAPKs and NF-κB signaling pathways, thereby playing a key role in innate immunity [32–35]. Accumulating evidence has also suggested that reactive oxygen species (ROS) function as important intermediaries, regulating various cellular signaling pathways in biological and physiological processes [36]. Our previous studies suggested that cytosolic ROS were essential in activating inflammatory responses during mycobacterial and toxoplasma infections [32, 35, 37, 38]. These studies strongly suggested that MAPKs and NF-κB signaling pathways and intracellular ROS generation contributed to the outcomes of inflammatory responses.

In the present study, we examined the anti-inflammatory role and molecular mechanisms of traditional herbal medicines, including *SdLv*, *Buch*, and *Bufa*, in primary macrophages infected with intracellular *Mab* or *T*. *gondii*. Moreover, we further investigated the function of *SdLv* in the *in vivo* infection mouse model of *Mab*.

## Materials and methods

### Mice and cell preparation

Wild-type (WT) C57BL/6 mice were purchased from Koatech (Pyungtek, Korea) and maintained in a pathogen-free environment condition. Bone marrow-derived macrophages (BMDMs) were isolated from femurs of mice and differentiated for 5–7 days in medium containing macrophage colony-stimulating factor (25 μg/ml, R&D Systems Minneapolis, MN, USA), as described previously [35]. The culture medium consisted of Dulbecco's modified Eagle's medium (DMEM, Life Technologies, Grand Island, NY, USA) supplemented with 10% heat-inactivated fetal bovine serum (FBS, Gibco BRL, Grand Island, NY, USA), 1 mM sodium pyruvate, 50 U/mL penicillin, 50 μg/mL streptomycin, and 5 × 10^−5^ M β-mercaptoethanol. The human retinal pigment epithelial cell lines ARPE-19 was purchased from the American Type Culture Collection (ATCC, Manassas, VA, USA) and grown in DMEM supplemented with 10% FBS or with nutrient mixture F-12, 10% FBS and 1% Antibiotic-Antimycotic (Gibco BRL, Grand Island, NY, USA). ARPE-19 cells were passaged by 0.25% Trypsin-EDTA (Life Technologies, Carlsbad, CA, USA) every 2–3 days.

### Preparation of the *Buch*, *Bufa* and *SdLv*

The roots of *Bupleurum chinense* DC., *Bupleurum falcatum* L. and *Stellaria dichotoma* var. *lanceolata* Bunge were purchased from Kwangmyeongdang Medicinal Herbs Co. (Ulsan, Korea). All samples were authenticated by evaluation of their microscopic and macroscopic characteristics by Dr. Goya Choi of the Korea Institute of Oriental Medicine (KIOM). The voucher specimens were deposited in the Korean Herbarium of Standard Herbal Resources at KIOM. (Index herbariorum code KIOM, Specimen No. 2-16-0424 (*Buch*), 2-16-0383 (*Bufa*), 2-16-0191(*SdLv*). *Buch* (779.31 g), *Bufa* (798.38 g) and *SdLv* (793.33 g) were extracted twice with 70% (v/v) ethanol using a 2 h reflux extraction at 80°C and then concentrated under reduced pressure. The 70% ethanol extracts were filtered through a standard sieve, evaporated to dryness, and freeze-dried. The yields were 12.21% *Buch*, 20.63% *Bufa* and 41.86% *SdLv*. The percentage yield was calculated by dividing the mass of product obtained (g) by the mass of sample (g). Prior to use, the lyophilized powders were dissolved in 0.25% sodium carboxymethyl cellulose (CMC; for *in vivo* analysis) or 0.01% DMSO (for *in vitro* analysis).

### Preparation of *Mab* and *T*. *gondii*

*Mab* ATCC 19977 strain was obtained from ATCC (Manassas, VA, USA) and cultured as previously described [32]. Briefly, *Mab* was grown for 4 days, at 37°C on Middlebrook 7H9 broth (Difco, Sparks, MD, USA) supplemented with oleic acid-albumin-dextrose-catalase (OADC, Becton Dickinson, Sparks, MD, USA) and 0.05% Tween 80 (Sigma-Aldrich, St. Louise, MO, USA) and stored at −70°C until used. The number of viable bacteria on Middlebrook 7H10 agar (Difco, Sparks, MD, USA) was determine as colony-forming units (CFU).

Tachyzoites of *T*. *gondii* RH strain were maintained ARPE-19 cells for 2 or 3 days at 37°C, 5% CO2 and biweekly passaged in DMEM with 10% FBS, nutrient mixture F-12, antibiotics. To collect *T*. *gondii* tachyzoites, cell debris including ARPE-19 cells and parasites were washed in cold phosphate-buffered saline (PBS) and then resuspended in cold culture medium. The suspension was passed through a 27-gauge needle and a 5 μm pore size polycarbonate membrane (Millipore, Bedford, MA, USA) to remove host cells.

### Reagents and antibody

Specific antibodies against phospho-ERK1/2 (9101), phospho-p38 (9211), phospho-SAPK/JNK (9251), IκBα (9242), and phospho-IKKα/β (2697) were purchased from Cell Signaling. Specific antibodies against β-tubulin (ab6046) were purchased from Abcam. All other reagents were purchased from Sigma-Aldrich, unless otherwise indicated.

### Ethics statement

Animal experimental procedures were approved by the Institutional Animal Care and Use Committee (IACUC) at Chungnam National University (CNU-00706) and at Chungnam National University Hospital (CNUH-017-A0012) and conformed to National Institutes of Health guidelines. Animal husbandry was provided by the staff of the IACUC under the guidance of supervisors who are certified Animal Technologists, and by the staff of the Animal Core Facility. Veterinary care was provided by IACUC faculty members and veterinary residents located on the Chungnam National University School of Medicine. The animals were fed standard rodent food and water ad libitum, and housed (maximum of 5 per cage) in sawdust-lined cages in an air-conditioned environment with 12-hour light/dark cycles. To minimize pain or illness of *Mab*-infected mice, the irreversible condition leading the inevitable death, such as loss of body condition, failure to drink, abnormal breathing, blood in the feces, was monitored daily and each mouse were euthanized with carbon dioxide [39].

### Cell counting kit (CCK) 8 assay

The cytotoxicity effects of herbal extracts on BMDMs were determined using CCK 8 assay (Dojindo Molecular Technologies, CK04-11) according to manufacturer’s protocol. Briefly, BMDMs were seeded in 96-well plates and differentiated with M-CSF for 5 days as described in cell preparation. Cells were replaced with serum-free media and then incubated with various herbal extracts for 24 hours. Then, 10 μl of the CCK-8 solution was added and incubated for 1 hours at 37°C. Absorbance was measured at 450 nm using a microplate reader (SPECTRO star Nano, BMG Labtech, Ortenberg, Germany).

### Experimental *in vivo* infection models

For *in vivo* experiments, we designed two model based on the infection rate of *Mab*. 1) Mice were orally administrated with *SdLv* (200 mg/kg) for 4 days, consecutively and then intravenously (i.v.) infected with *Mab* (5 × 10^8^ CFU/mouse). On the 1 day after infection of *Mab*, mice were further administrated with *SdLv* for 3 days, consecutively. On the 4 day after infection of *Mab*, mice were euthanized, and bacterial burden and mRNA expression of *Tnf* were analyzed in the lungs and spleens, respectively. (2) Mice were orally administrated with *SdLv* (200 mg/kg) for 4 days, consecutively and then intravenously (i.v.) infected with *Mab* (5 × 10^9^ CFU/mouse). On the 1 day after infection of *Mab*, mice were further administrated with *SdLv* for 7 days, consecutively. The body weight and survival of each mouse were monitored for 12 days. For a negative control, 0.25% CMC was orally administered instead of *SdLv*.

### RNA extraction, real-time quantitative PCR, semi-quantitative RT-PCR, Western blot analysis, and enzyme-linked immunosorbent assays (ELISAs)

RNA extraction, real-time quantitative PCR, and semi-quantitative RT-PCR were performed as described previously [40]. The sequences of the primers used were as follows: mTNFα (forward: 5′-AGCACAGAAAGCATGATCCG-3′; reverse: 5′-CTGATGAGAGGGAGGCCATT-3), mIL-6 (forward: 5′-GGAAATTGGGGTAGGAAGGA-3′; reverse: 5′-CCGGAGAGGA-GACTTCACAG -3′), mIL-1β (forward: 5′-CTCCATGAGCTTTGTACAAGG-3′; reverse: 5′-TGCTGATGTACCAGTTGGGG-3′), mIL-12p40 (forward: 5′-GACCATCACTGTCAAAGAGTT-3′; reverse: 5′-AGGAAAGTCTTGTTTTTGAAA-3′), and mβ-actin (forward: 5′-TCATGAAGTG TGACGTTGACATCCGT-3′; reverse: 5′-CCTAGAAGCATTTGCGGTGCACGATG-3′).

For Western blot analysis, cell lysates were collected and lysed in PRO-PREP (iNtRON BIOTECHNOLOGY, Korea) containing additional set of phosphatase inhibitors. Protein concentration was determined using a BCA assay kit. Proteins (30 μg/each conditions) were immediately heated for 5 min at 100°C. Each sample was subjected to SDS-PAGE on gel containing 12% (w/v) acrylamide under reducing conditions. Separated proteins were transferred to PVDF membranes (Millipore Corp., Billerica, MA, USA), and then the membranes were blocked with 5% skim milk. Membranes were developed using chemiluminescence assay kit (ECL; Millipore Corp., Billerica, MA, USA) and subsequently analyzed using Chemiluminescence Imaging System (Davinch-K, Seoul, Korea). Data were analyzed using Alliance Mini HD6 (UVitec Cambridge, MA, USA).

In the sandwich ELISA, cell culture supernatants were analyzed using DuoSet antibody pairs (BD Pharmingen) for the detection of mouse tumor necrosis factor (TNF)-α and interleukin (IL)-6.

### Measurement of ROS production

Level of intracellular ROS was determined by dihydroethidium (DHE, Calbiochem), 2′,7′-dichlorodihydroflurescein (DCFDA, Invitrogen), or CellROX Green reagent (Thermo Fisher Scientific, Waltham, MD, USA) [37]. Briefly, cells were incubated with 10 μM DHE for 30 min, 20 μM H2DCFDA for 30 min, 1 μM CellROX for 30 min at 37 xC and then analyzed by a confocal microscope (LSM 710; Zeiss), fluorescence microscope (SZX7; Olympus) or a FACS Calibur flow cytometer (Becton-Dickinson, San Josè, CA, USA).

### Statistical analyses

Differences between averages were analyzed by a two-tails paired Student’s *t*-test with Bonferroni adjustment or log-rank test in survival and are presented as the means ± SD. Statistical comparisons were carried out using GraphPad Prism software (GraphPad Software, Inc. La Jolla, CA, USA). Differences were considered significant at *p* <0.05.

## Results

### *SdLv* has no cytotoxic effects on primary macrophages

**Fig 1:**
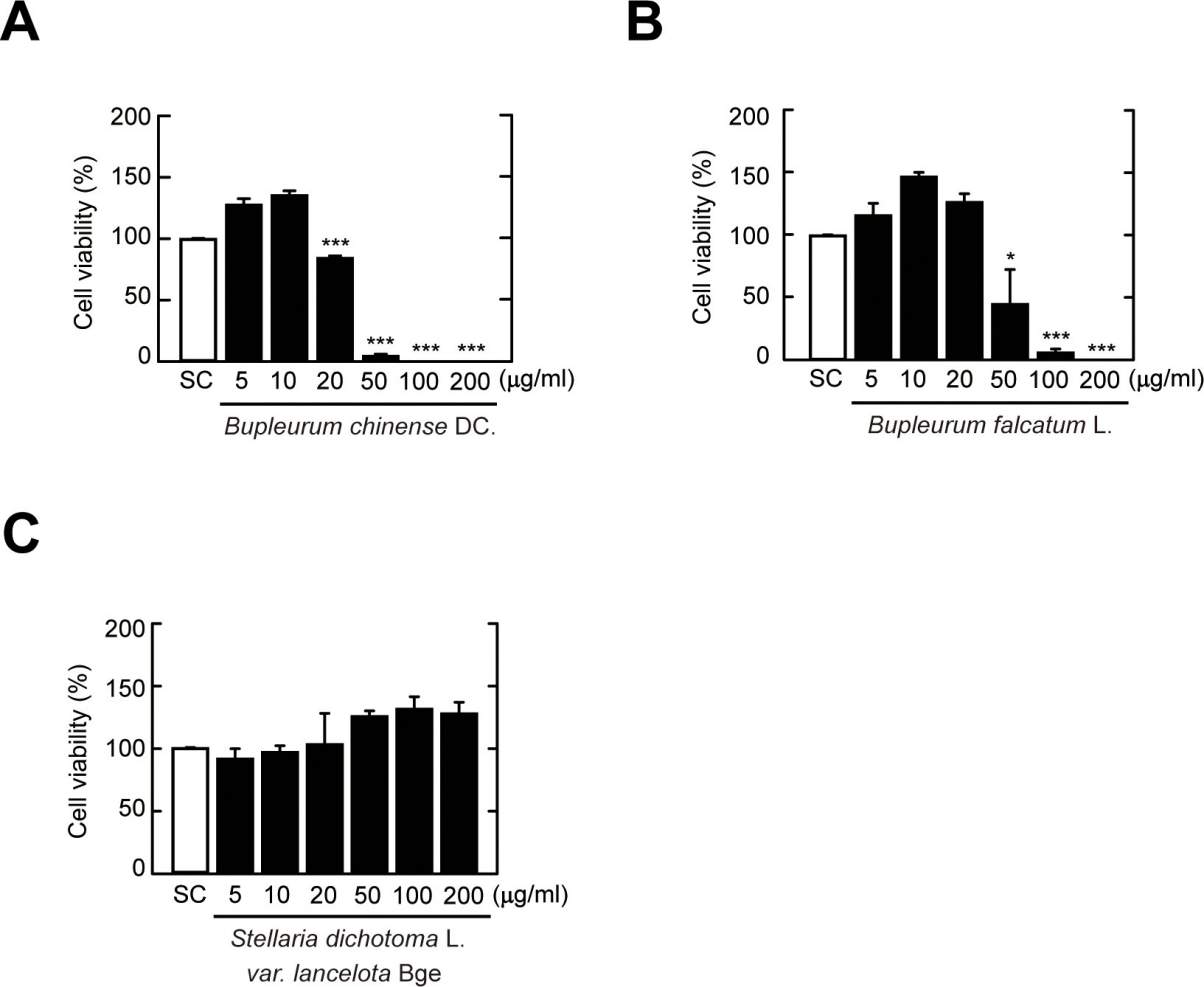
Cytotoxic effects of herbal extracts in BMDMs. (A-C) BMDMs were treated with different concentrations of each herbal extract (5–200 μg/ml) for 24 h, and cell viability was evaluated using CCK-8 assay. Quantitative data for cell viability are representative of three independent experiments and are presented as means ± SD. *P < 0.05, ***P < 0.001 (two-tailed Student’s t-test), compared with SC-treated cells. SC, vehicle control (0.01% DMSO).

To investigate the host protective roles of herbal extracts against intracellular pathogens, we first determined the cytotoxic effects of three candidates, including *SdLv*, *Bufa*, and *Buch*, in BMDMs. As shown in Fig 1, the stimulation of BMDMs with *SdLv* had no significant effect on either cell viability at various concentrations (5–200 μg/mL) over 24 hours (Fig 1C) or on solvent control-stimulated cells. However, cell viability was significantly decreased after treatment with 20 μg/mL *Buch* (Fig 1A) or 50 μg/mL *Bufa* (Fig 1B). Based on these results, we used concentrations of *SdLv* (5–200 μg/ml), *Bufa* (5–20 μg/ml), and *Buch* (5–10 μg/ml) that did not exert a cytotoxic effect.

### *SdLv* attenuates *Mab*-induced generation of proinflammatory cytokines in primary macrophages

**Fig 2:**
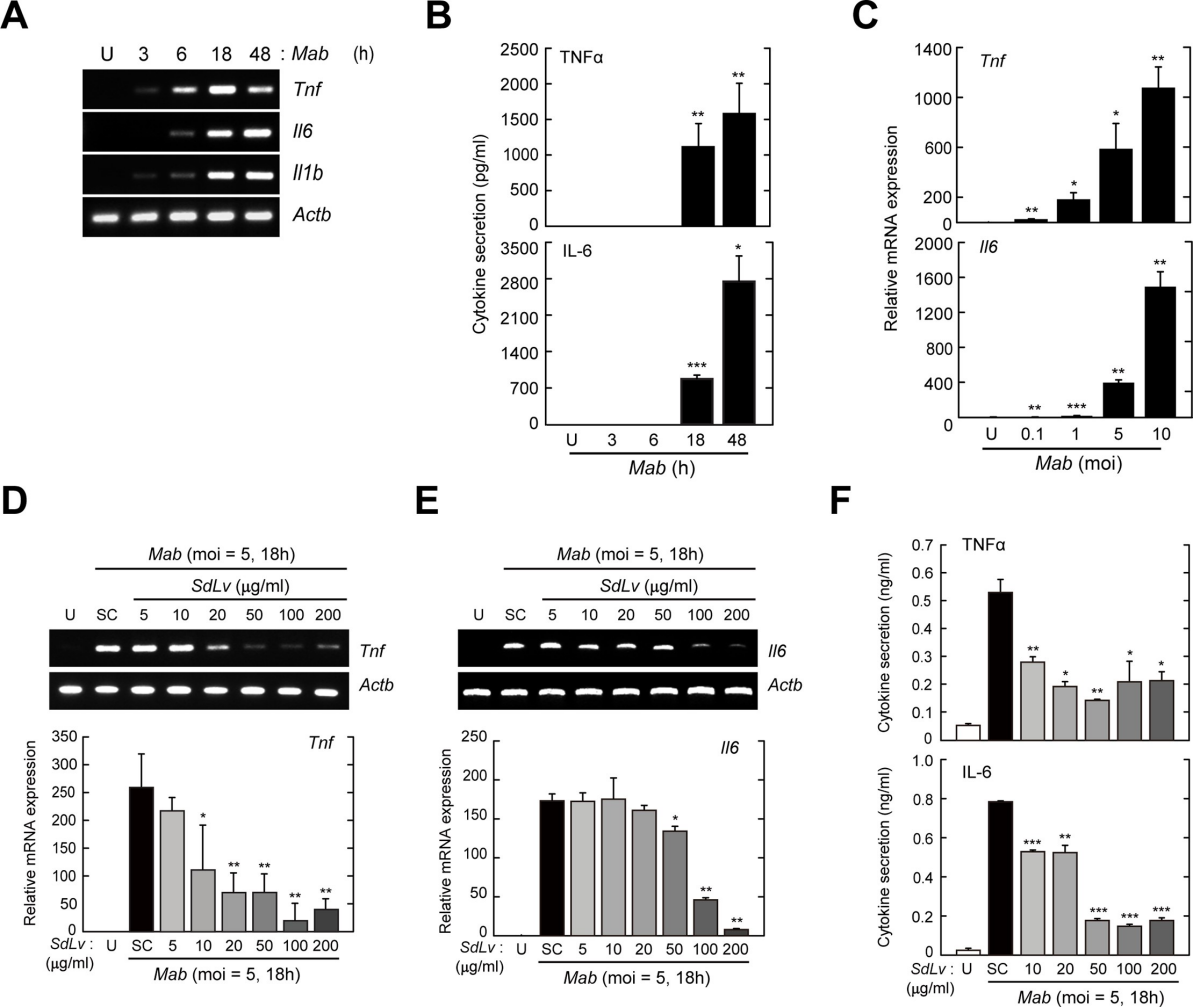
*SdLv* inhibits *Mab*-mediated the generation of TNF-α and IL-6 in BMDMs. (A and B) BMDMs were infected with *Mab* (MOI = 5) for the indicated time periods (A) Cell lysates was collected and the mRNA expression of *Tnf*, *Il6* and *Il1b* then measured using semiquantitative RT-PCR analysis. *Actb* (encoding β-actin) serves as a loading control throughout. (B) Culture supernatant was collected and the generation of TNF-α and IL-6 protein then were measured using ELISA assay. (C) BMDMs were infected with *Mab* (at MOI = 0.1, 1, 5 or 10) for 18 h. semiquantitative RT-PCR analysis of *Tnf* and *Il6* mRNA. (D—F) BMDMs were stimulated with increasing concentration of *SdLv* (1 h, 5–200μg/ml), followed by *Mab* infection (MOI = 5) for 18 h. (D and E) Semi-quantitative RT-PCR (top) or Quantitative RT-PCR analysis (bottom) were assessed to evaluate the mRNA expression of *Tnf* (for D) and *Il6* (for E). (F) Each culture supernatant was collected and the production of TNF-α and IL-6 were measured using ELISA assay. Data are representative of three independent experiments and are presented as means ± SD. *P < 0.05, **P < 0.01, ***P < 0.001 (two-tailed Student’s t-test), compared with uninfected cells (B and C) or *Mab*-infected control cells (D—F). U, Untreated; SC, vehicle control (0.01% DMSO).

Based on the previous studies showing that alkaloid constituents from *SdLv* and exhibited anti-inflammatory [4] and anti-allergic effects [41], we examined the effects of herbal extracts, including *SdLv*, *Bufa*, and *Buch*, on the *Mab*-mediated activation of inflammatory responses. As shown in Fig 2A and 2B, we infected BMDMs with *Mab* for various time periods and then assessed the mRNA (Fig 2A) and protein (Fig 2B) expressions of inflammatory cytokines, such as TNF-α and IL-6. We also assessed the mRNA expression levels of the *Tnf* and *Il6* genes at 18 hours after the infection of *Mab* in a MOI-dependent manner (Fig 2C).

**Fig 3:**
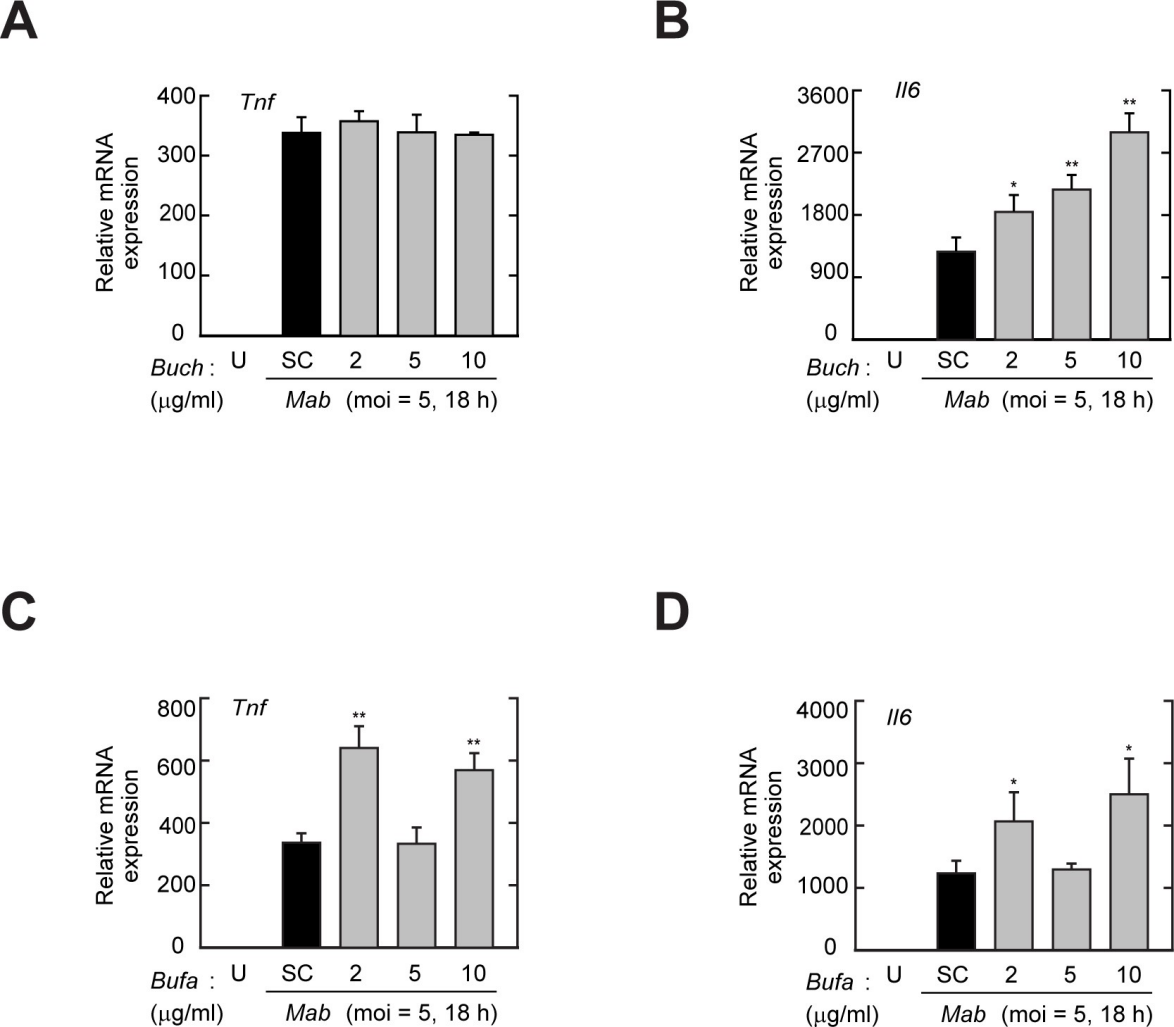
*Mab*-mediated the inflammatory response is not attenuated by pretreatment of *Buch* and *Bufa*. (A and B) BMDMs were pretreated with *Buch* (2, 5, or 10μg/ml) for 1 h and then infected 18 h with *Mab* (MOI = 5). Total RNAs were extracted and subjected to quantitative RT-PCR analysis to measure the mRNA expression of *Tnf* (for A) and *Il6* (for B). (C and D) BMDMs were pretreated with *Bufa* (2, 5, or 10μg/ml) for 1 h and then infected 18 h with *Mab* (MOI = 5). Total RNAs were extracted and subjected to quantitative RT-PCR analysis to measure the mRNA expression of *Tnf* (for C) and *Il6* (for D). Data are representative of three independent experiments and are presented as means ± SD. *P < 0.05, **P < 0.01 (two-tailed Student’s t-test), compared with *Mab*-infected control cells. U, Untreated; SC, vehicle control (0.01% DMSO).

To determine whether herbal extracts, including *SdLv*, *Bufa*, and *Buch*, inhibited the *Mab*-mediated generation of inflammatory cytokines, BMDMs were infected with *Mab* in the presence or absence of each herbal extract, and the expressions of TNF-α and IL-6 were evaluated by RT-PCR, qPCR, or an ELISA (Fig 2D–2F and Fig 3A–3D). *Mab*-induced mRNA expression of the *Tnf* (Fig 2D) and *Il6* (Fig 2E) genes were significantly attenuated by pretreatment with *SdLv* in a concentration-dependent manner. Moreover, the secretions of TNF-α and IL-6 in culture supernatants after *Mab* infection were also decreased by pretreatment with *SdLv* in a concentration-dependent manner (Fig 2F). However, these inhibitory effects were not observed in the presence of *Buch* (Fig 3A and 3B) or *Bufa* (Fig 3C and 3D); rather, *Mab*-induced mRNA expressions of the *Tnf* and *Il6* genes were significantly increased under some conditions when pretreated with *Buch* and *Bufa*. These results indicated that *SdLv*, but not *Bufa* and *Buch*, regulated *Mab*-mediated inflammatory responses.

### *T*. *gondii*-mediated inflammatory responses are not altered by pretreatment of primary macrophages with *SdLv*

Because innate immune recognition of *T*. *gondii*, a well-known intracellular parasite, promotes host immunity and inflammatory responses similar to those of mycobacterial infection [42, 43], we determined the effects of *SdLv* in regulating the *T*. *gondii-*induced activation of proinflammatory responses in primary macrophages. We first evaluated the mRNA (S1A Fig) or protein (S1B Fig) expression levels of proinflammatory cytokines in BMDMs infected with *T*. *gondii* for the indicated time periods. We also further assessed the mRNA expression levels of the *Tnf* and *Il6* genes at 48 hours after the infection of *T*. *gondii* in a MOI-dependent manner (S1C Fig).

Because *SdLv* has anti-inflammatory properties during *Mab* infection, we identified its roles in *T*. *gondii* infection. BMDMs were infected with *T*. *gondii* in the presence or absence of *SdLv*, and the expressions of TNF-α and IL-6 were evaluated by RT-PCR, qPCR, or ELISA (S1D–S1F Fig). As shown in S1D and S1E Fig, pretreatment of *SdLv* did not affect *T*. *gondii*-induced mRNA expression of the *Tnf* and *Il6 genes* compared with *T*. *gondii*-infected BMDMs pretreated with the solvent control. Moreover, the secretions of TNF-α and IL-6 in culture supernatants after *T*. *gondii* infection were also not altered by pretreatment with *SdLv* (S1F Fig). Together, these results indicated that *SdLv*, unlike infection with *Mab*, had no effects on the regulation of the inflammatory responses of *T*. *gondii* infection.

### *SdLv* modulates the activation of c-Jun N-terminal kinase (JNK) and the p38 MAPK pathway in response to *Mab* but not to *T*. *gondii*

**Fig 4:**
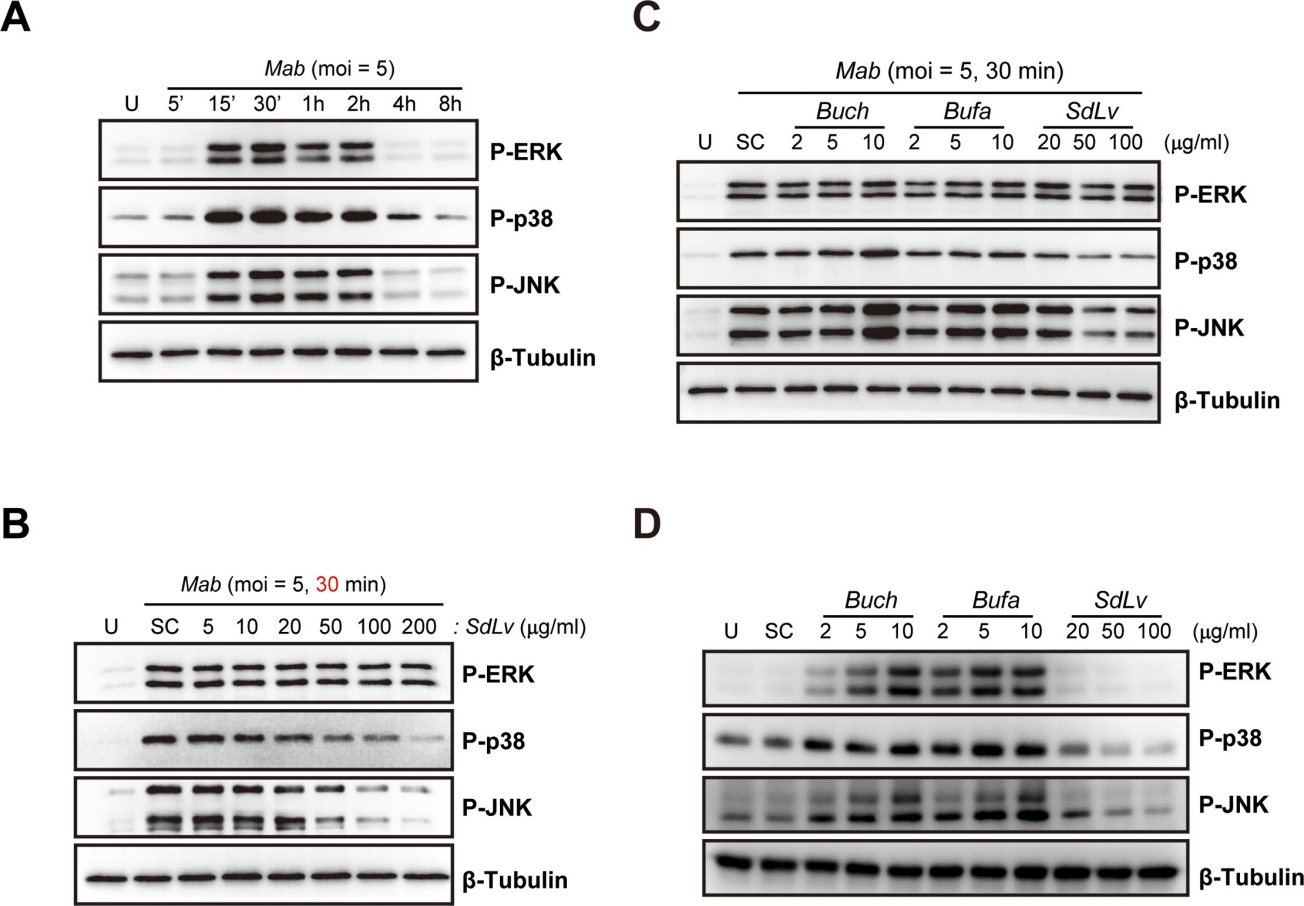
*SdLv* regulates *Mab*-induced the activation of JNK and p38 MAPK in BMDMs. (A) BMDMs were infected with *Mab* (MOI = 5) for the indicated time periods. (B) BMDMs were infected with *Mab* (MOI = 5, 30 min) in the presence or absence of *SdLv* (5–200 μg/ml). (C) BMDMs were pretreated with *Buch* (2, 5, or 10 μg/ml), *Bufa* (2, 5, or 10 μg/ml), or *SdLv* (20, 50, or 100 μg/ml) for 1 h and subsequently infected with *Mab* (MOI = 5, 30 min). (D) BMDMs were treated with *Buch* (2, 5, 10 μg/ml), *Bufa* (2, 5, 10 μg/ml), or *SdLv* (20, 50, 100 μg/ml) for 1 hours. MAPKs activation was determined by western blotting analysis for phosphorylated ERK, p38, and JNK/SAPK antibody. Total protein was determined by monitoring β-tubulin as a loading control. Representative data from one of three independent experiments with similar results are shown. U, Untreated; SC, vehicle control (0.01% DMSO).

MAPKs are known to be essential kinases for the formation of transcription factor complex AP-1, which is involved in the activation of inflammatory responses upon diverse microbial infections [30, 44]. We therefore examined whether *SdLv* affected the MAPK signaling pathways activated by the infection of *Mab* or *T*. *gondii*. Primary macrophages infected with *Mab* (Fig 4A) or *T*. *gondii* (S2A Fig) showed rapid activation of all three MAPK subfamilies, including p38, extracellular signal-regulated kinases (ERK) 1/2, and JNK, within 30 minutes. Notably, *Mab*-mediated phosphorylation of p38 and JNK, but not ERK 1/2, were attenuated by pretreatment with *SdLv* in a concentration-dependent manner (Fig 4B). We next examined whether *SdLv* regulated the activation of three MAPK subfamilies in response to *T*. *gondii* infection in a similar manner as *Mab* infection. As shown in S2B Fig, *T*. *gondii*-mediated phosphorylation of p38, ERK 1/2, and JNK were not modulated by pretreatment with *SdLv* in BMDMs. We also found that *Mab*-mediated activations of these kinases were not attenuated by pretreatment with *Bufa* and *Buch*, but was rather enhanced in *Mab*-infected BMDMs pretreated with *Buch* (10 μg/ml) and *Buch* (5 and 10 ug/ml) (Fig 4C). Furthermore, we found that BMDMs treated with *Buch* or *Buch* showed the enhanced phosphorylation of ERK 1/2, p38, and JNK in a dose-dependent manner, whereas *SdLv* has no effects (Fig 4D). Taken together, these findings indicated that *SdLv* may selectively inhibit the *Mab*-mediated activation of inflammatory responses through regulation of the p38 and JNK signaling pathways.

### *SdLv* attenuates the activation of the *Mab*-induced NF-κB signaling pathway in macrophages

**Fig 5:**
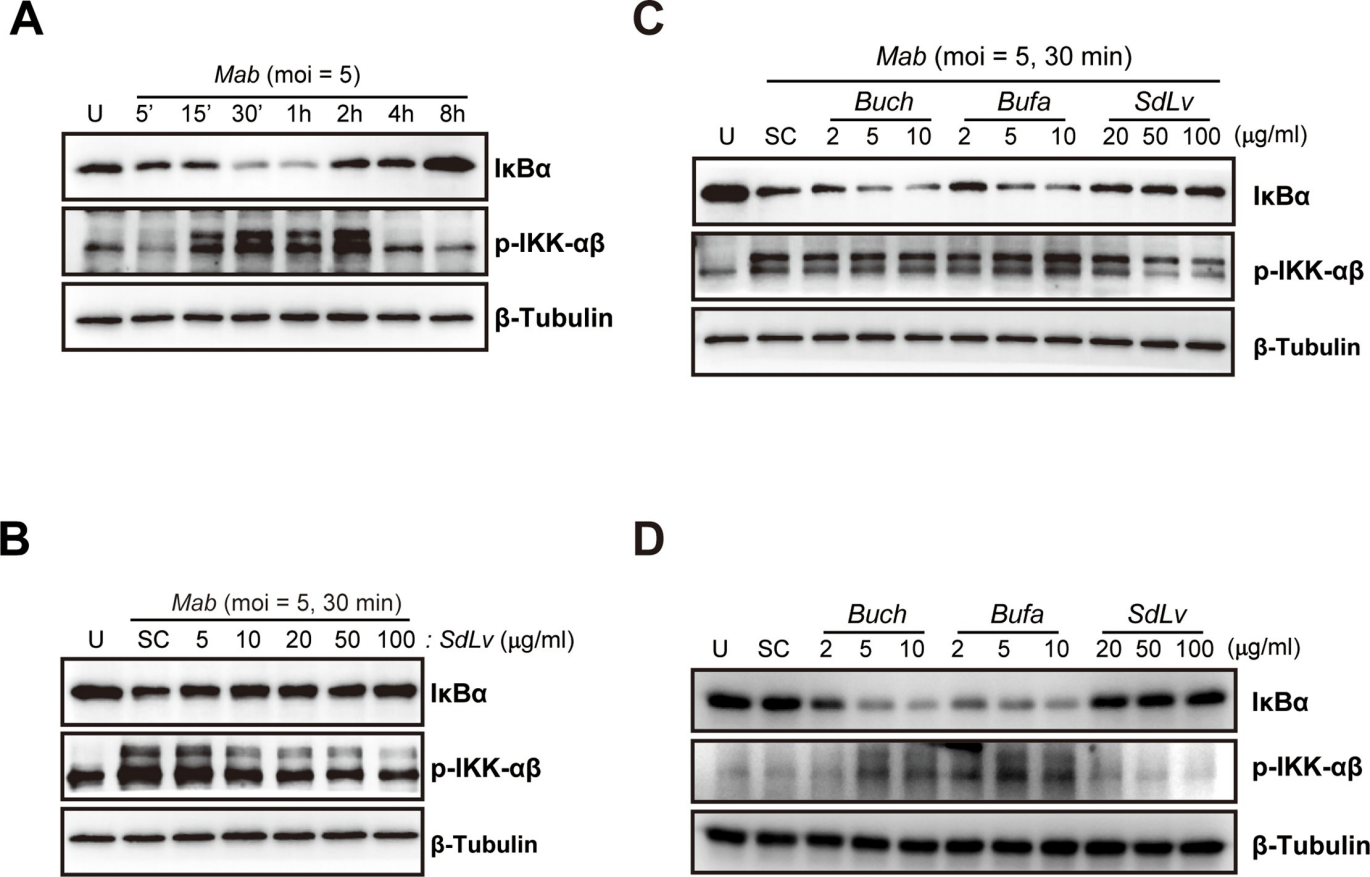
*SdLv*, but not *Buch* and *Bufa*, effectively attenuates the activation of NF-κB signaling in response to *Mab*. (A) BMDMs were infected with *Mab* (MOI = 5) for the indicated time periods. (B) BMDMs were infected with *Mab* (MOI = 5, 30 min) in the presence or absence of *SdLv* (5–200 μg/ml). (C) BMDMs were pretreated with *Buch* (2, 5, or 10 μg/ml), *Bufa* (2, 5, or 10 μg/ml), or *SdLv* (20, 50, or 100 μg/ml) for 1 h and subsequently infected with *Mab* (MOI = 5, 30 min). (D) BMDMs were treated with *Buch* (2, 5, 10 μg/ml), *Bufa* (2, 5, 10 μg/ml), or *SdLv* (20, 50, 100 μg/ml) for 1 hours. Cell lysate were collected and protein expression of phosphorylated IKKαβ and total IκBα were determined by western blotting analysis. Total protein was determined by monitoring β-tubulin as a loading control. Representative data from one of three independent experiments with similar results are shown. U, Untreated; SC, vehicle control (0.01% DMSO).

NF-κB signaling is activated by the stimulation of diverse pathogens, including bacterial, viral, fungal, and parasitic agents, and is closely associated with the activation of inflammatory responses [44]. We showed that infection with *Mab* (Fig 5A) and *T*. *gondii* (S2C Fig) resulted in the rapid activation of the kinases, IKKα and IKKβ, leading to the degradation of the NF-κB inhibitor, IκB-α. To examine the inhibitory roles of *SdLv* in *Mab*-induced activation of NF-κB signaling, BMDMs were infected with *Mab* for 30 minutes in the presence of *SdLv*, and phosphorylation of IKKα/β and degradation of IκB-α were then evaluated using immunoblot assays. As shown in Fig 5B, pretreatment with *SdLv* in *Mab*-infected BMDMs effectively attenuated the activation of NF-κB signaling. However, these inhibitory effects were not only observed in *Bufa*- and *Buch*-pretreated cells, but were also activated in some conditions pretreated with *Bufa* and *Buch* (Fig 5C). Similar to those shown in MAPK signaling pathways (Fig 4D), the stimulation of *Bufa* or *Buch*, but not *SdLv*, resulted in the degradation of IκB-α and the phosphorylation of IKKαβ in primary murine macrophages (Fig 5D). We next examined the effects of *SdLv* in *T*. *gondii*-induced activation of NF-κB signaling expression. Similar to MAPKs signaling, *T*. *gondii*-mediated phosphorylation of IKKαβ and degradation of IκB-α were not attenuated by *SdLv* pretreatment of BMDMs (S2D Fig). Taken together, these results showed that *SdLv* effectively attenuated *Mab*-mediated activation of the NF-κB signaling pathway, which may be crucial for the regulation of inflammation.

### *SdLv* regulates *Mab*-induced generation of intracellular reactive oxygen species (ROS) in macrophages

**Fig 6:**
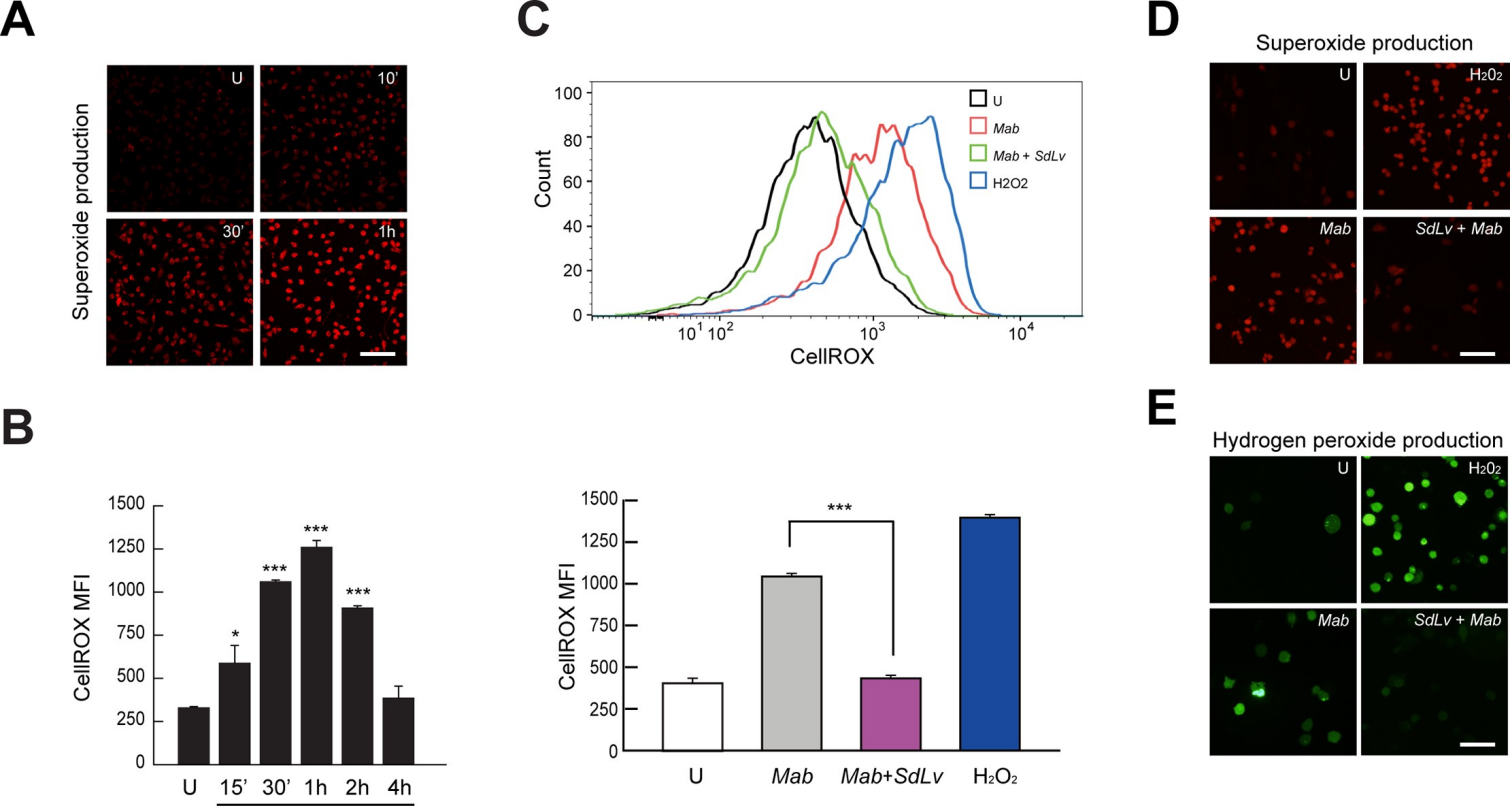
*Mab*-induced intracellular ROS generation is attenuated by pretreatment of *SdLv* in BMDMs. (A) BMDMs were infected with *Mab* (MOI = 5) for indicated time periods and were then stained with DHE (10 μM) for 30 min to measure intracellular superoxide using confocal microscopy. (scale bars = 50 μm) (B) BMDMs were infected with *Mab* (MOI = 5) for indicated time periods and were then stained with CellROX (1 μM) for 30 min to measure intracellular oxidative stress using flow cytometry. (C-E) BMDMs were infected with *Mab* (MOI = 5) for 30 min in a presence or absence of *SdLv*. H2O2 (1 mM) was used for positive control. (C) Cells were stained with CellROX (1 μM) for 30 min and intracellular oxidative stress were measured using flow cytometry. (D-E) Cells were stained with DHE (10 μM) for 30 min (scale bars = 50 μm for D) or H_2_DCFDA (20 μM) for 30 min (scale bars = 25 μm for E) to measure superoxide or hydrogen peroxide using fluorescence microscope, respectively. Data represent the means and SD of three independent experiments. *p < 0.05, ***p < 0.001 (two-tailed Student’s t-test), compared with uninfected cells (B) or *Mab*-infected cells (C). U, Untreated.

Previous studies have reported that intracellular ROS are required for the activation of inflammatory responses as a signaling intermediate [35, 38, 45, 46]. Moreover, Lim et al., reported that enzyme extracts from *Stellaria dichotoma* had antioxidant properties [6]. We thus assessed the generation of intracellular ROS using oxidized DCFDA (for hydrogen peroxide), DHE (for superoxide anion), and CellROX (for detection of general oxidative stress) using flow cytometry, fluorescence microscopy, or laser-based confocal microscopy, respectively. Exposure of BMDMs to *Mab* resulted in rapid generation of intracellular superoxide within 10–30 minutes, with peak activation at 1 hour after infection (Fig 6A), with an increase of cellular oxidative stress (Fig 6B). Additionally, pretreatment with *SdLv* effectively attenuated the *Mab*-mediated activation of cellular oxidative stress (Fig 6C), intracellular superoxide production (Fig 6D), and intracellular hydrogen peroxide production (Fig 6E). However, the stimulation of *Buch* and *Bufa* mediated the significant increase of cellular oxidative stress in BMDMs. In addition, *Mab*-induced the activation of cellular oxidative stress was slightly increased in the presence of *Buch* or *Bufa* (S3 Fig). Together, these results indicated that *Mab*-induced activation of oxidative stress was attenuated by *SdLv*.

### *SdLv* inhibits proinflammatory responses and increases survival of an *in vivo* mouse infection model of *Mab*

**Fig 7:**
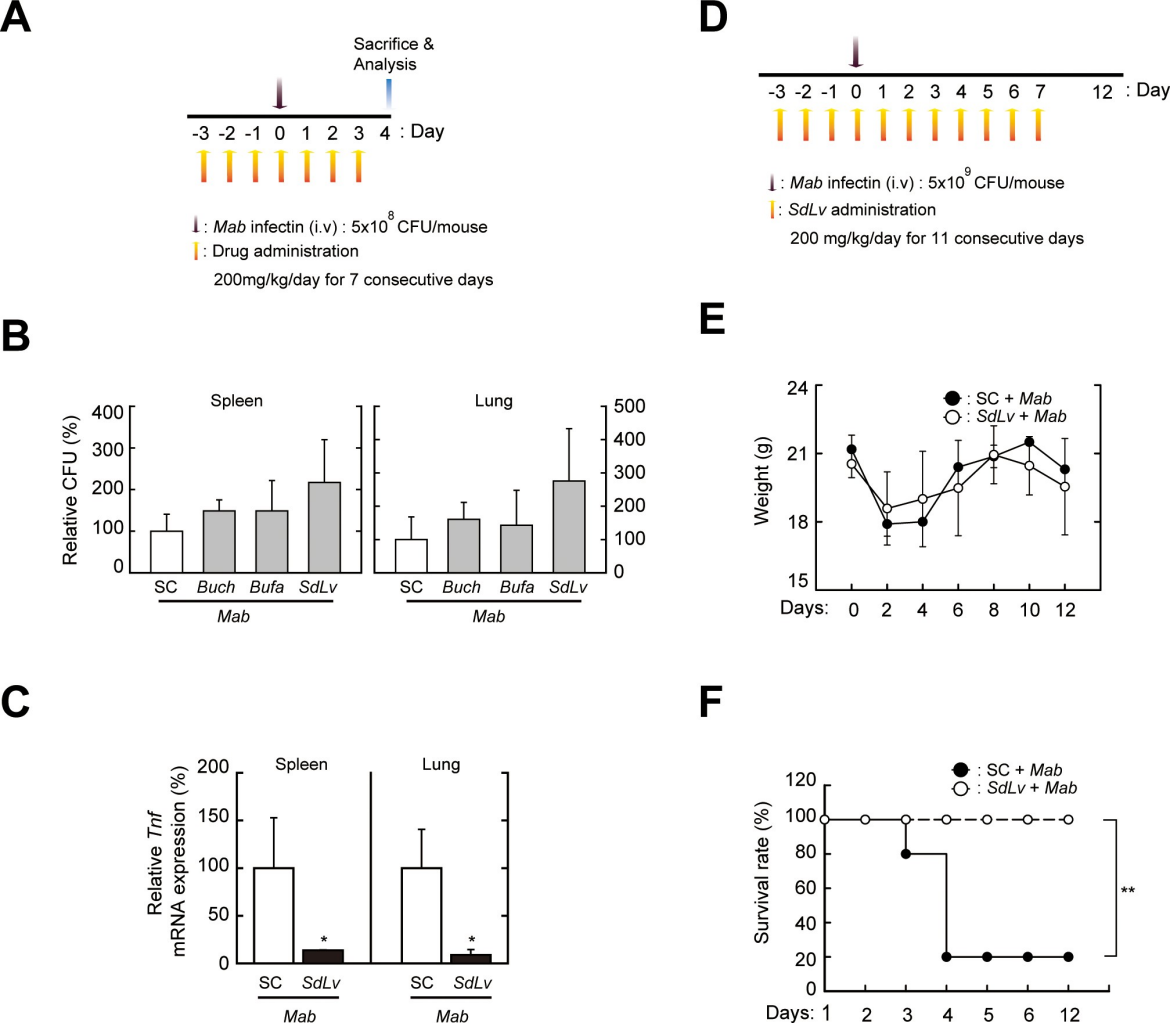
*SdLv* contributes to the host protection through the regulation of proinflammatory responses *in vivo* infection models of *Mab*. (A-C) Mice received each herbal extracts, such as *Buch*, *Bufa*, or *SdLv* (200 mg/kg; administrated orally) or a solvent control for 7 consecutive days and then infected with *Mab* (5 × 10^8^ CFU/mouse, i.v.) at 3 days after first drug administration. The mice were sacrificed at 4 days after the infection of *Mab*. (A) *In vivo* experimental schedule to determine bacterial loads (for B) and *Tnf* mRNA expression (for C) in tissues. (B) *In vivo* mycobacterial burden of infected mice (n = 5 per group) in spleen (left) and liver (right) were determined by CFU assay. (C) *Tnf* mRNA expression in spleen and liver were evaluated using qPCR (n = 3 per group). (D-F) Mice received *SdLv* (200 mg/kg; administrated orally) for 11 consecutive days and then infected with *Mab* (5 × 10^9^ CFU/mouse, i.v.) at 3 days after first drug administration. (D) *In vivo* experimental schedule. (E and F) The body weight (for D) or survival rate (for E) of each mouse (n = 5 per group) were monitored for 12 days. Data are presented as mean ± SD. *p < 0.05, **p < 0.01, compared with control mice infected with *Mab* (two-tailed Student’s t-test (C) or log-rank test (F)). SC, vehicle control (0.25% CMC). CFU, colony-forming units.

To investigate the *in vivo* efficacy of *SdLv*, we used an *Mab* mouse intravenous infection model [47, 48]. As described in the Material and Methods section and Fig 7A and 7D, the mice that received *SdLv* (200 mg/kg) for 4 days were intravenously injected with *Mab* [5 × 10^8^ CFU/mouse (Fig 7A–7C) or 5 × 10^9^ CFU/mouse (Fig 7D–7F)] and then further treated with *SdLv* for the indicated time periods. As shown in Fig 7B, the bacterial burden in the spleen and lung tissues were not significantly altered in *SdLv*-administered mice. However, the *Tnf* mRNA expressions in the spleen and lung tissue of each mouse were significantly attenuated by the administration of *SdLv* (Fig 7C). Based on the anti-inflammatory roles of *SdLv in vivo*, we further determined the body weight (Fig 7E) and survival rate (Fig 7F) in each mouse infected by a high dose of *Mab* (5 × 10^9^ CFU/mouse), as described in Fig 7D. As shown in Fig 7E and 7F, there was no change in the body weight between each experimental group (Fig 7E); however, the lethality associated with the infection of *Mab* was dramatically decreased in *SdLv*-administered mice compared with in solvent control-treated mice. Together, these *in vivo* findings indicated that *SdLv* improved the survival of mice through the regulation of *Mab*-induced inflammatory responses but not through bacterial clearance.

## Discussion

To date, various efforts have been made to identify the function of major components and to characterize the underlying mechanisms to improve treatments for inflammatory disorders and infectious diseases. In East Asia, a variety of herbal extracts, including *SdLv*, *Bufa*, and *Buch*, have been used to treat fever-related illnesses, such as influenza and malaria [3, 8, 10]. Notably, diverse compositions including saikosaponin, fatty acids and essential oil in *Bupleurum* species and flavonoids, sterols, cyclic peptides, and β-carboline-type alkaloids in *Stellaria dichotoma* are important for their biological activities [6, 49]. Although recent studies have shown that these extracts possess diverse biological and physiological properties, the functions of these herbal extracts in mycobacterial or toxoplasma infection and their mechanisms of action have yet to be fully elucidated. In this study, we identified the effects of herbal medicines on the innate immune response to *Mab* infection in primary macrophages and *in vivo* in a mouse model.

Increasing evidence has shown that the interaction between innate immune cells and diverse pathogens rapidly trigger inflammation, thereby playing a crucial role in the activation of host protective immunity. Furthermore, an aberrant activation of inflammation is responsible for immunopathogenesis in various infectious or non-infectious diseases [44]. The *Mab* complex causes pulmonary disease in patients with cystic fibrosis, which is closely associated with the disruption of balance between inflammation and tissue remodeling during repeated bacterial infections [23, 50, 51]. Although TNF-α is an essential cytokine that controls mortality and bacterial growth during mycobacterial infection [52, 53], excessive production of TNF-α results in the impaired survival of mice despite sufficient bacterial clearance [54]. These studies suggested that the optimal regulation of inflammatory responses may be responsible for the improvement of mycobacteria-associated diseases. In this study, we found that *SdLv* effectively attenuated the *Mab*-induced mRNA and protein expression of proinflammatory cytokines in primary murine macrophages. Consistent with this finding, *in vivo* administration of *SdLv* significantly reduced the mRNA expression of TNF-α in the spleen and lung tissues of *Mab*-infected mice, although the bacterial burden in each tissue was not altered by *SdLv* treatment. Moreover, the lethality in *Mab*-infected mice was significantly improved by treatment with *SdLv*. These findings indicated that *SdLv* contributes to host protection against *Mab* infection by the regulation of inflammatory responses rather than by mycobacterial killing.

During mycobacterial infection, different PRRs recognize bacteria and/or bacterial-derived components and then activate intracellular signals leading to the generation of inflammatory cytokines and the initiation of adaptive immune responses [55]. Previous studies showed that MAPKs signaling pathways are crucial for the activation of inflammatory responses through toll like receptor (TLR) 2 in response to *Mab* [32, 33]. Additionally, during myeloid differentiation primary response gene 88 (*MyD88*) is also required for the production of TNF-α in BMDMs [33]. Consistent with these findings, *M*. *abscessus* subsp. *massiliense* (*M*. *mas*), which belongs to the *Mab* complex, induced the generation of TNF-α and IL-6 through MyD88- and JNK-dependent signaling pathways in murine macrophages [56]. In the present study, we found that *SdLv* specifically attenuated *Mab*-mediated activation of the JNK and p38 MAPK pathways but not of the ERK 1/2 pathways. However, *SdLv* did not inhibit *T*. *gondii*-induced production of inflammatory cytokines or activation of the three MAPK subfamilies. It would have been of interest if the inhibitory roles of *SdLv* had differed depending on the type of bacteria and protozoa. In acute toxoplasmosis, the hyperactivation of the inflammatory responses is mediated by extensive liver damage and lymphoid degeneration, which are closely related to lethality in mice [57]. The infection of the high virulence type 1 RH strain of *T*. *gondii* induced the activation of p38 MAPK and IL-12 production via a MyD88-independent manner in BMDMs [58]. Although we could not extend our findings to the role of MyD88-dependent inflammatory responses, our findings indicated the crucial role of *SdLv* in the regulation of inflammatory responses in response to *Mab* but not to *T*. *gondii*, which was mediated by the inhibition of the JNK and p38 MAPK signaling pathways in macrophages.

Stimulation of mycobacteria and their components induces the production of inflammatory cytokines through the NF-κB signaling pathway as well as the MAPK signaling pathway, which are crucial for the activation of innate and adaptive immunities [59]. Previous studies reported that *Mab* infection resulted in the translocation of NF-κB p65 into the nuclei [33, 48]. Moreover, the *M*. *mas*-induced NF-κB signaling pathway was required for the production of TNF-α and IL-6 in BMDMs [56]. Here, we found that *SdLv* inhibited the degradation of IκBα and the phosphorylation of IKKα/β in *Mab*-infected BMDMs.

Numerous studies have reported that intracellular ROS act as signaling molecules and play essential roles in regulating a broad range of biological responses [60]. Previous studies also reported that intracellular ROS generation was required for the production of proinflammatory cytokines via TRAF6-mediated activation of the ASK1-p38 pathway in response to TLR4 [61]. Moreover, ROS regulated TLR4-mediated IL-8 expression via a NF-κB signaling pathway in the human monocyte/macrophage cell line, THP-1 [62]. In a similar manner, intracellular ROS are also involved in the activation of the inflammatory response in *M*. *bovis* bacille Calmette-Guerin- or *M*. *tuberculosis*-infected macrophages [37, 38]. Moreover, Dectin-1-dependent activation of spleen tyrosine kinase is crucial for *Mab*-induced intracellular ROS generation and the production of inflammatory cytokines in BMDMs [32]. In response to *M*. *mas*, NADPH oxidase 2-induced ROS modulated the activation of the inflammatory response via the JNK-dependent signaling pathway [56]. Together, our data strongly support that *SdLv* plays an important role in the regulation of *Mab*-mediated intracellular ROS generation and it is thought to be effective against the infection of various atypical mycobacteria.

In the present study, we found that *Mab* infection induced the generation of inflammatory cytokines in primary murine macrophages, which was significantly attenuated by pretreatment with *SdLv*, but not *Bufa* or *Buch*. To elucidate the reasons for this disparity in the roles of these herbal extracts in *Mab* infection, we evaluated their cytotoxicity and effect on the activity of the intracellular signaling pathways that regulate the inflammatory response. The *Bufa* and *Buch* extracts had stronger cytotoxic effects at lower concentrations compared to the *SdLv* extract. Moreover, treatment for 1 h with *Bufa* or *Buch*, but not *SdLv*, led to activation of the NF-κB and MAPK signaling pathways and the generation of intracellular ROS. In future studies we plan to identify the active components that mediate cytotoxic and inflammatory effects in these compounds.

In summary, we identified novel functions of *SdLv* in regulating *Mab*-induced inflammatory responses by targeting the generation of ROS and the subsequent activation of MAPKs and NF-κB signaling pathways in macrophages. Additionally, *SdLv* had protective effects against *Mab* through the regulation of excessive inflammation in the spleen and lung tissues of mice. Our findings suggested the efficacy of *SdLv* not only against other mycobacterial infections but also for a variety of inflammatory diseases, including sepsis and inflammatory bowel disease, which are closely associated with uncontrolled oxidative stress. Because crude extracts isolated from *SdLv* contain diverse bioactive compounds, further studies should therefore identify the most effective compounds purified from herbal extracts, which can be used in the regulation of inflammatory responses and to evaluate possible clinical applications for the control of mycobacterial infections.

## Supporting information

S1 Fig *SdLv* do not compromise *T*. *gondii*-mediated inflammatory responses in primary macrophages. (A and B) BMDMs were infected with *T*. *gondii* (MOI = 1) for the indicated time periods (A) Cell lysates was collected and the mRNA expression of *Tnf*, *Il6*, *Il1b* and *Il12p40* then measured using semiquantitative RT-PCR analysis. *Actb* (encoding β-actin) serves as a loading control throughout. (B) Culture supernatant was collected and the generation of TNF-α and IL-6 protein then were measured using ELISA assay. (C) BMDM were infected with *T*. *gondii* (at MOI = 0.1, 1, 5 or 10) for 18 h. semiquantitative RT-PCR analysis of *Tnf* and *Il6* mRNA. (D—F) BMDMs were stimulated with increasing concentration of *SdLv* (1 h, 5–200μg/ml), followed by *T*. *gondii* (MOI = 1) for 18 h. (D and E) Semi-quantitative RT-PCR (top) or quantitative RT-PCR analysis (bottom) were assessed to evaluate the mRNA expression of *Tnf* (for D) and *Il6* (for E). (F) Each culture supernatant was collected and the production of TNF-α and IL-6 were measured using ELISA assay. Data are representative of three independent experiments and are presented as means ± SD. *P < 0.05, **P < 0.01, ***P < 0.001 (two-tailed Student’s t-test), compared with uninfected cells (B and C). U, Untreated; SC, vehicle control (0.01% DMSO).
(TIF)

S2 Fig *T*. *gondii*-mediated the activation of MAPK and NF-κB signaling is not inhibited by the pretreatment of *SdLv* in primary murine macrophages. (A and C) BMDMs were infected with *T*. *gondii* (MOI = 1) for the indicated time periods. (B and D) BMDMs were infected with *T*. *gondii* (MOI = 1, 30 min) in the presence or absence of *SdLv* (A and B) Immunoblot analysis was performed to determine protein expression of phosphorylated ERK, p38, or JNK. β-tubulin served as a loading control. (C and D) Immunoblot analysis was performed to determine protein expression of total IκB-α and phosphorylated IKKα/β. Data are representative of three independent experiments. U, Untreated; SC, vehicle control (0.01% DMSO).
(TIF)

S3 Fig *Mab*-mediated the activation of intracellular oxidative stress is not inhibited by the pretreatment of *Buch* and *Bufa* in primary murine macrophages. BMDMs were treated with *Buch* or *Bufa* for 1 hours and then infected with or without *Mab* (MOI = 5) for 30 min. H2O2 (1 mM) was used for positive control. Cells were stained with CellROX (1 μM) for 30 min and intracellular oxidative stress were measured using flow cytometry. Data are representative of three independent experiments. U, Untreated; SC, vehicle control (0.01% DMSO).
(TIF)
