# Supplementary figures and images for: Protective effects of a traditional herbal extract from Stellaria dichotoma var. lanceolata against Mycobacterium abscessus infections
Source: PLoS One. 2018 Nov 19;13(11):e0207696. doi: 10.1371/journal.pone.0207696 (PMC6242687; doi:10.1371/journal.pone.0207696)

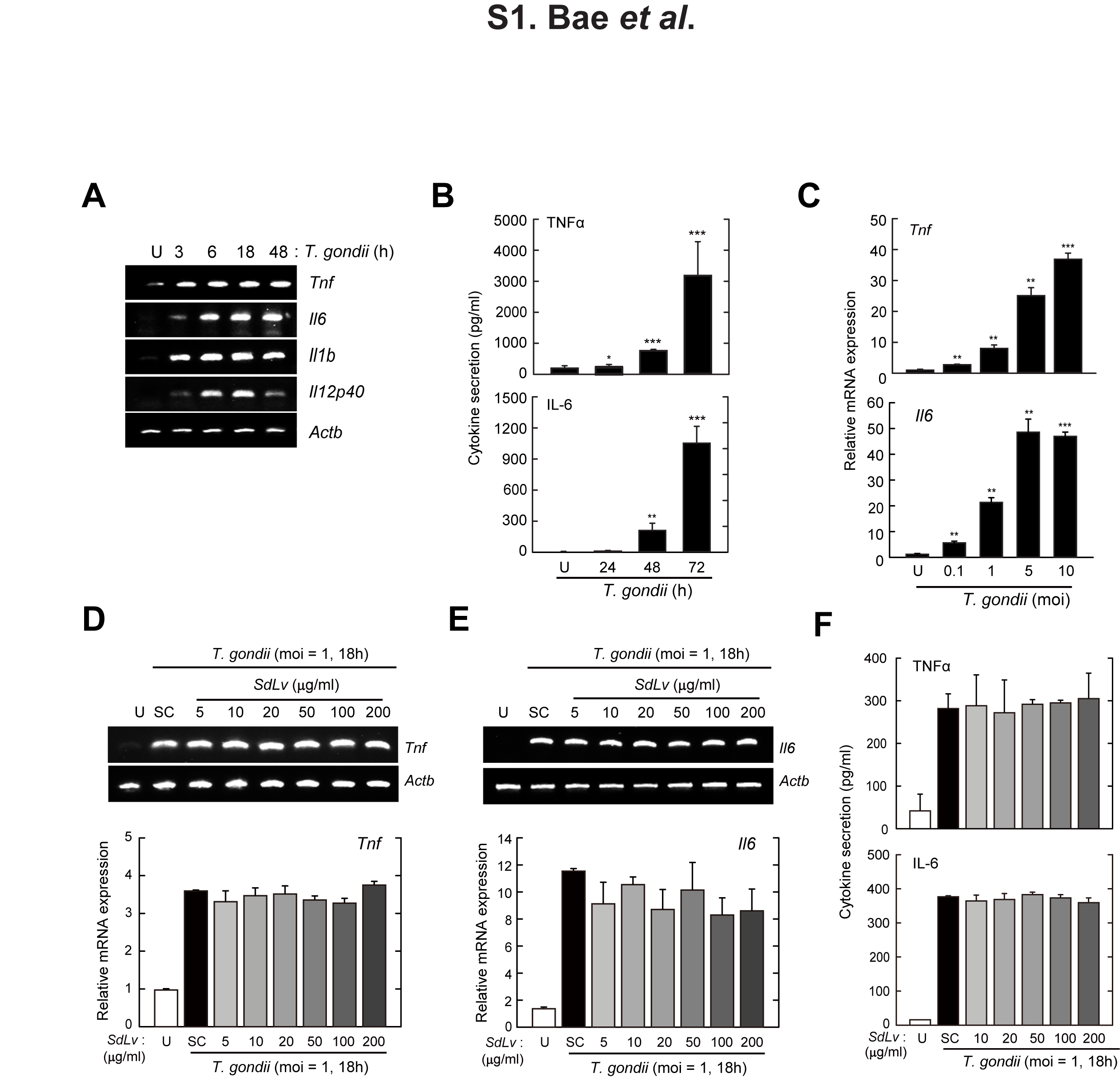

Supplement: S1 Fig — (A and B) BMDMs were infected with T. gondii (MOI = 1) for the indicated time periods (A) Cell lysates was collected and the mRNA expression of Tnf, Il6, Il1b and Il12p40 then measured using semiquantitative RT-PCR analysis. Actb (encoding β-actin) serves as a loading control throughout. (B) Culture supernatant was collected and the generation of TNF-α and IL-6 protein then were measured using ELISA assay. (C) BMDM were infected with T. gondii (at MOI = 0.1, 1, 5 or 10) for 18 h. semiquantitative RT-PCR analysis of Tnf and Il6 mRNA. (D—F) BMDMs were stimulated with increasing concentration of SdLv (1 h, 5–200μg/ml), followed by T. gondii (MOI = 1) for 18 h. (D and E) Semi-quantitative RT-PCR (top) or quantitative RT-PCR analysis (bottom) were assessed to evaluate the mRNA expression of Tnf (for D) and Il6 (for E). (F) Each culture supernatant was collected and the production of TNF-α and IL-6 were measured using ELISA assay. Data are representative of three independent experiments and are presented as means ± SD. *P < 0.05, **P < 0.01, ***P < 0.001 (two-tailed Student’s t-test), compared with uninfected cells (B and C). U, Untreated; SC, vehicle control (0.01% DMSO). (TIF) [file pone.0207696.s001.tif]

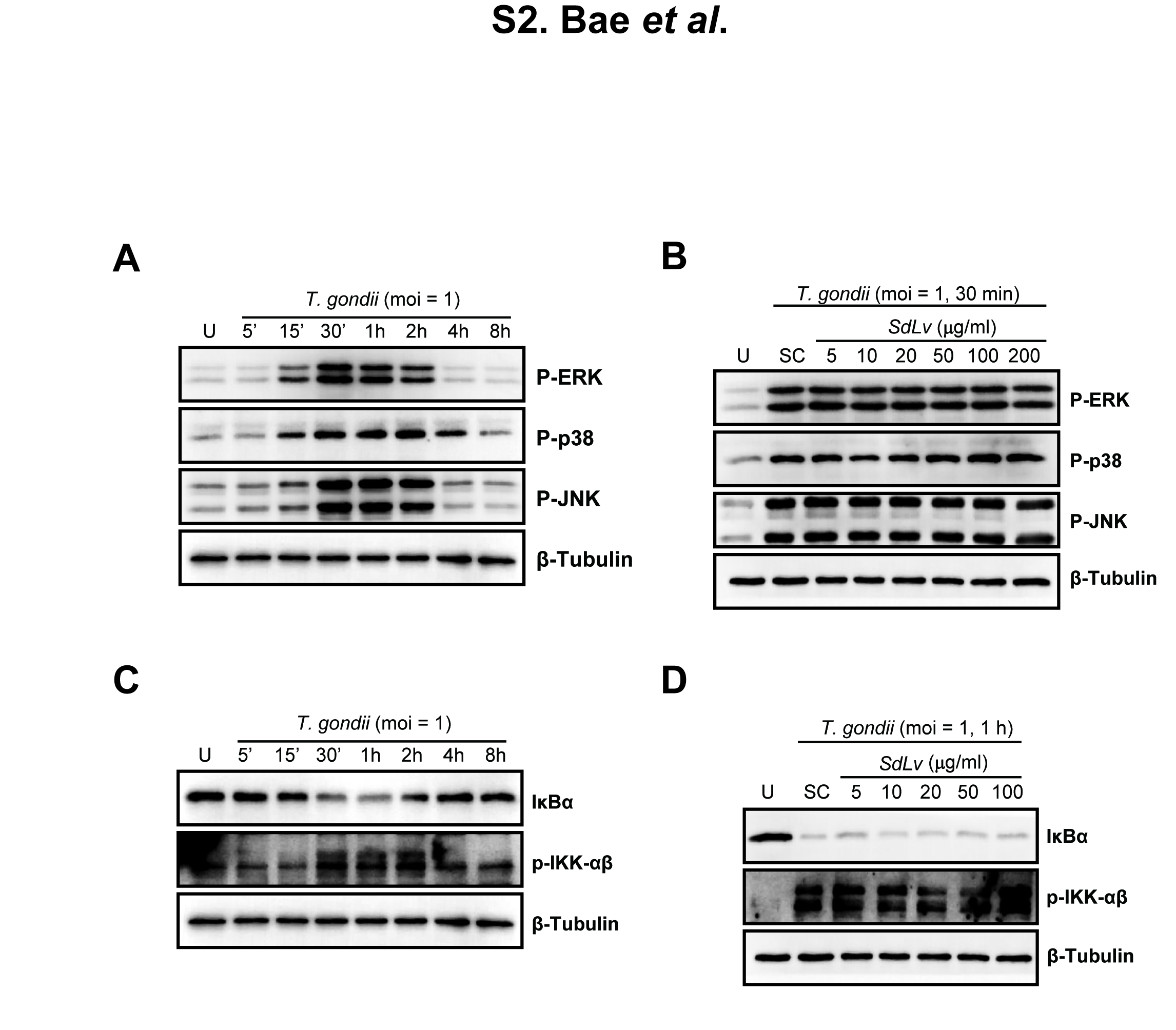

Supplement: S2 Fig — (A and C) BMDMs were infected with T. gondii (MOI = 1) for the indicated time periods. (B and D) BMDMs were infected with T. gondii (MOI = 1, 30 min) in the presence or absence of SdLv (A and B) Immunoblot analysis was performed to determine protein expression of phosphorylated ERK, p38, or JNK. β-tubulin served as a loading control. (C and D) Immunoblot analysis was performed to determine protein expression of total IκB-α and phosphorylated IKKα/β. Data are representative of three independent experiments. U, Untreated; SC, vehicle control (0.01% DMSO). (TIF) [file pone.0207696.s002.tif]

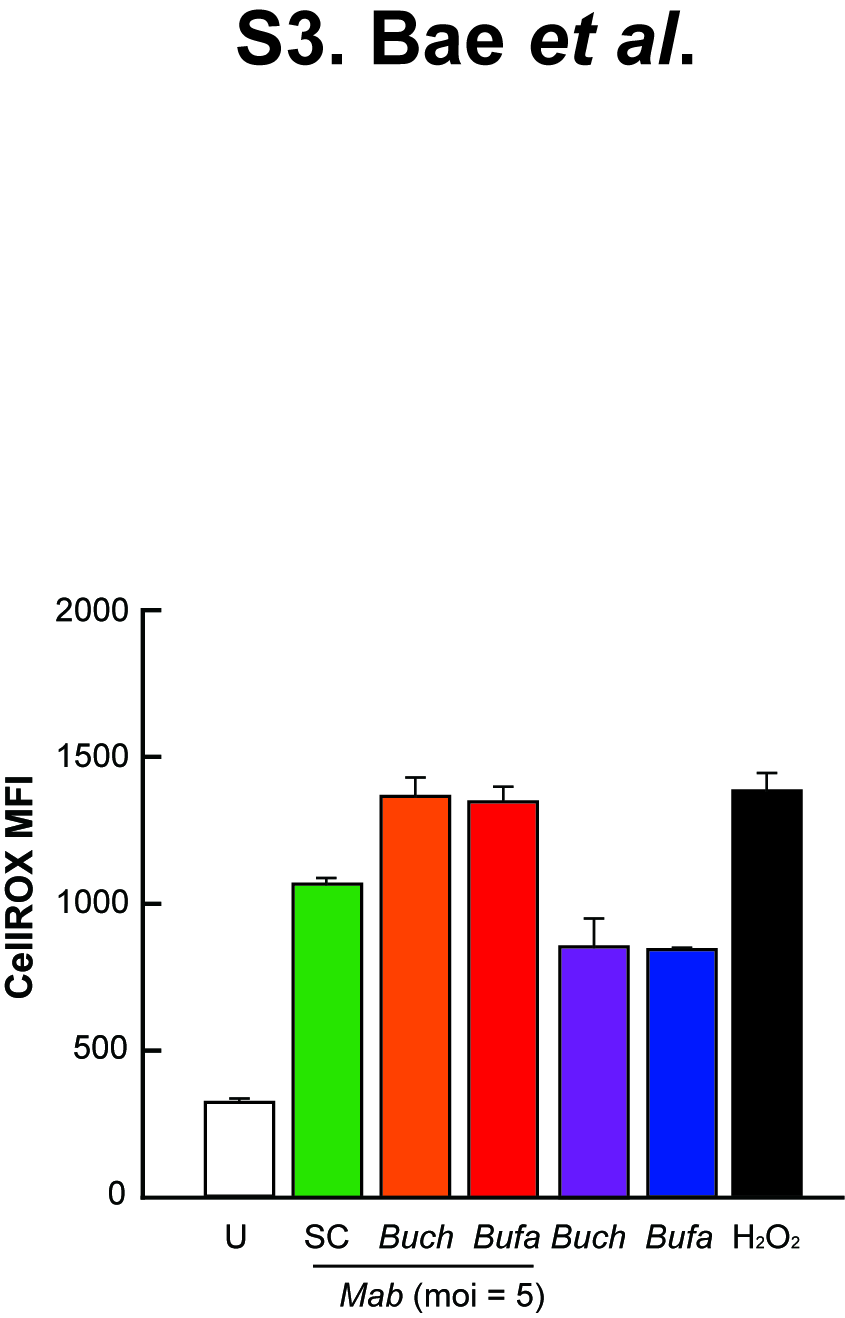

Supplement: S3 Fig — BMDMs were treated with Buch or Bufa for 1 hours and then infected with or without Mab (MOI = 5) for 30 min. H2O2 (1 mM) was used for positive control. Cells were stained with CellROX (1 μM) for 30 min and intracellular oxidative stress were measured using flow cytometry. Data are representative of three independent experiments. U, Untreated; SC, vehicle control (0.01% DMSO). (TIF) [file pone.0207696.s003.tif]
